# Draft Genome Sequence of a Multidrug-Resistant *Acinetobacter baumannii* PKAB07 Clinical Strain from India Belonging to Sequence Type 195

**DOI:** 10.1128/genomeA.00184-14

**Published:** 2014-03-20

**Authors:** Rajagopalan Saranathan, Archana Tomar, Pagal Sudhakar, K. P. Arunkumar, K. Prashanth

**Affiliations:** a Department of Biotechnology, School of Life Sciences, Pondicherry University, Pondicherry, India; b Laboratory of Molecular Genetics, Centre for DNA Fingerprinting and Diagnostics (CDFD), Hyderabad, India

## Abstract

*Acinetobacter baumannii* has emerged as one of the most common nosocomial pathogens and is considered to be a significant threat to public health worldwide. Here, we present the draft genome sequence of a multidrug-resistant clinical strain of *A. baumannii* PKAB07 isolated from a wound infection in India during 2011 to 2012.

## GENOME ANNOUNCEMENT

*Acinetobacter baumannii* is a major nosocomial pathogen causing severe infections in hospitalized patients worldwide. Recent reports document the emergence of multidrug-resistant (MDR) *A. baumannii* strains in the Indian subcontinent harboring carbapenem-hydrolyzing OXA-type carbapenemases and metallo-β-lactamases (1, 2). To date, whole-genome sequences of *A. baumannii* clinical strains isolated in India have not been available in GenBank. Here, we present for the first time the whole-genome draft sequence of an MDR clinical isolate of *A. baumannii* from India. This carbapenem-resistant isolate (*A. baumannii* PKAB07) was isolated from a wound infection in a hospitalized patient and identified through multilocus sequence type (MLST) analysis to be sequence type 195 (ST195).

Genomic paired-end reads of 2 × 100 nucleotides were generated on an Illumina Genome Analyzer II instrument. A total of around 9.4 million reads of insert size between 180 and 220 bp were generated. The adapter sequences were trimmed from the reads before assembly using the FASTX-Toolkit (http://hannonlab.cshl.edu/fastx_toolkit/). *De novo* assembly of trimmed sequences was carried out with three different assembly tools: Velvet (3), in conjunction with the VelvetOptimiser (http://bioinformatics.net.au/software.velvetoptimiser.shtml), ABySS (4), and the A5 pipeline (5). Velvet produced 152 contigs using a *k*-mer length of 75. ABySS generated 155 contigs using a *k*-mer of 75. The A5 pipeline yielded 60 contigs using the default parameters. All the contigs from different assemblies were pooled, and contigs of <200 nucleotides were filtered. A total of 284 contig sequences were reordered against 15 currently available complete genome sequences of *A. baumanni* using Mauve (6). The highest level of similarity was observed against the *A. baumanni* ACICU genome, which was used as a reference sequence by abacas.pl (http://abacas.sourceforge.net/index.html) to generate a genome assembly of 4,233,806 bp. The assembled genome annotation was conducted with the RAST automated annotation engine. Resistance-related genes and insertion sequences were analyzed using ResFinder and IS Finder, respectively.

Around 3,877 protein-coding genes, including 59 genes related to resistance to antibiotics and toxic compounds, as well as 102 RNA genes were predicted by the RAST server. The NCBI Prokaryotic Genome Annotation Pipeline predicted around 3,821 protein-coding genes and 80 tRNA genes. ResFinder identified resistance genes encoding aminoglycoside-modifying enzymes (*armA*, *strA*, and *strB*), β-lactamases (*bla*_OXA-66_ and *bla*_ADC-1_), macrolide resistance (*mphE* and *msrE*), and tetracycline resistance (*tetB*). IS Finder found IS*Aba1* to be present in 26 copies throughout the genome and IS*Aba22* in a single copy. Twenty-one rRNA genes were recognized to be present (7 each of 5S, 16S, and 23S rRNAs) through RNammer analysis. Resistance islands were predicted through G+C content analysis using the GC-Profile tool.

A detailed report on the analysis of the genome data in the context of resistance and virulence characteristics will be given in future publications. This announcement makes the *A. baumannii* PKAB07 genome data available for further analysis to understand its pathogenic potential.

### Nucleotide sequence accession numbers.

This whole-genome shotgun project has been deposited in DDBJ/EMBL/GenBank under accession no. CP006963 and CP006964. The version described in this paper is the ﬁrst version.

## References

[1] Uma Karthika R Srinivasa Rao R Sahoo S Shashikala P Kanungo R Jayachandran S Prashanth K 2009 Phenotypic and genotypic assays for detecting the prevalence of metallo-β-lactamases in clinical isolates of *Acinetobacter baumannii* from a South Indian tertiary care hospital. J. Med. Microbiol. 58:430–435. 10.1099/jmm.0.002105-0 19273637

[2] Karunasagar A Maiti B Shekar M Shenoy M S Karunasagar I 2011 Prevalence of OXA-type carbapenemase genes and genetic heterogeneity in clinical isolates of *Acinetobacter* spp. from Mangalore, India. Microbiol. Immunol. 55:239–246. 10.1111/j.1348-0421.2011.00313.x 21244471

[3] Zerbino DR Birney E 2008 Velvet: algorithms for *de novo* short read assembly using de Bruijn graphs. Genome Res. 18:821–829. 10.1101/gr.074492.107 18349386 PMC2336801

[4] Birol I Jackman SD Nielsen CB Qian JQ Varhol R Stazyk G Morin RD Zhao Y Hirst M Schein JE Horsman DE Connors JM Gascoyne RD Marra MA Jones SJ 2009 *De novo* transcriptome assembly with ABySS. Bioinformatics 25:2872–2877. 10.1093/bioinformatics/btp367 19528083

[5] Tritt A Eisen JA Facciotti MT Darling AE 2012 An integrated pipeline for *de novo* assembly of microbial genomes. PLoS One 7:e42304. 10.1371/journal.pone.0042304 23028432 PMC3441570

[6] Darling AC Mau B Blattner FR Perna NT 2004 Mauve: multiple alignment of conserved genomic sequence with rearrangements. Genome Res. 14:1394–1403. 10.1101/gr.2289704 15231754 PMC442156

